# Deploying a clinical innovation in the context of actor-patient consultations in general practice: A prelude to a formal clinical trial

**DOI:** 10.1186/1471-2288-9-54

**Published:** 2009-07-17

**Authors:** Moyez Jiwa, Robert K McKinley, Katrina Spilsbury, Hayley Arnet, Marthe Smith

**Affiliations:** 1 Western Australian Centre for Cancer and Palliative Care, Curtin Health Innovation Research Institute, Curtin University of Technology, Perth, Western Australia, Australia; 2 Keele University Medical School, Keele University, Staffordshire, UK; 3 Department of Public Health, Curtin University of Technology, Perth, Western Australia, Australia

## Abstract

**Background:**

Innovations to be deployed during consultations with patients may influence the clinical performance of the medical practitioner. This study examined the impact on General Practitioners' (GPs) consultation performance of novel computer software, designed for use while consulting the patient.

**Methods:**

Six GPs were video recorded consulting six actor-patients in a simulated clinical environment. Two sessions were recorded with six consultations per GP. Five cases presented cancer symptoms which warranted a referral for specialist investigation. Practitioners were invited to use a novel software package to process referrals made during the consultations in the second session. Two assessors independently reviewed the consultation performance using the Leicester Assessment Package (LAP). Inter-rater agreement was assessed by a Bland-Altman plot of the difference in score against the average score.

**Results:**

Sixty of the seventy two consultations were successfully recorded. Each video consultation was scored twice by two assessors leaving 120 LAP scores available for analysis. There was no evidence of a difference in the variance with increasing score (Pitmans test p = 0.09). There was also no difference in the mean differences between assessor scores whether using the software or not (T-test, P = 0.49)

**Conclusion:**

The actor-patient consultation can be used to test clinical innovations as a prelude to a formal clinical trial. However the logistics of the study may impact on the validity of the results and need careful planning. Ideally innovations should be tested within the context of a laboratory designed for the purpose, incorporating a pool of practitioners whose competencies have been established and assessors who can be blinded to the aims of the study.

## Background

Innovations are required in clinical practice in order to improve outcomes for patients. Implementation of such innovations requires substantial evidential support. Glazious and Haynes postulated that a significant barrier to the introduction of research evidence into clinical practice is that the relevant action must be recalled at the right time and the necessary task achievable at the opportune moment. [1] In many countries the general practitioner (GP) is one of the first health practitioners to consult patients with any significant health care problem. The core activity which takes place in general practice is the consultation. The function of the consultation has remained unchanged over many decades and the description by the British Royal College of General Practitioners in 1972 still applies today:

*"...the ideal consultation. The doctor's attention is devoted exclusively for a short period of time to the life and problems of another human being. He is there to listen and to help. His training will have made him receptive to a wide range of distress signals and given him the means, to answer them. The occasion will be unhurried and something will be gained by both participants; a good consultation brings satisfaction to the doctor as well as to the patient." *[2]

Therefore any intervention that may impact on this consultation has implications for outcome for the patient. Whilst there is a demand for more research to take place in general practice there is an on-going imperative to continue to function efficiently and effectively whether or not testing innovations or new ways of working. The perceived adverse impact on practitioner work flow may be one barrier to innovation in general practice.[3] Therefore any innovation must be tested for safety in respect to the impact on practitioner performance before being deployed in the field as a research or clinical tool.

### Theoretical framework

General Practitioners are required to manage multiple tasks during consultations. Meyer et al. describe two stages that help people to unconsciously switch between tasks. Human "executive control" processes have two distinct, complementary stages. [4] One is "goal shifting" ("I want to do this now instead of that"), the other "rule activation" ("I'm turning off the rules for *that *and turning on the rules for this"). Problems arise when switching costs conflict with productivity and safety, both of which are required in general practice. Thus, diagnostic and therapeutic errors may occur when either stage is compromised.

Although switch costs may be relatively small, they add up when switching repeatedly back and forth between tasks. Brief mental blocks created by shifting between tasks can consume up to 40% percent of someone's productive time and increase the risk of error. [5] It would be prudent to seek evidence that an innovation or research tool for use during the consultation does not disrupt that consultation and diminish the practitioners' competencies. Therefore the impact of the innovation must be studied within the consultation context before deploying that innovation in practice or recommending a formal clinical trial. We replicated the conditions of clinical practice and standardized, insofar as possible, the presenting problem to different practitioners in this experiment. We aimed to explore the impact on GP consultation performance of a new and unfamiliar computer program designed for use in the process of referring a patient to a specialist. Our objective was to compare performance on three of the six LAP categories of consultation competence (interviewing and history taking, problem solving and patient management) which were assessed in this study before and after the implementation of software designed to help make a written referral to a specialist.

## Methods

### Ethics

This study received ethics approval from HREC at Curtin University of Technology (RD-54-08)

### Design

This pre and post study involved two sessions of simulated consultations using actors as patients. These sessions were conducted on the premises of a General Practice in Perth, Western Australia.

### Actor-patients

Five of six 'patients' presented with a red flag symptom of common cancers. [6] The symptoms were readily recognizable as those of a cancer with a detailed history. The cases were reviewed by a team of clinicians working locally and in each case the need for a specialist consultation was endorsed. The team was general practitioners and specialist members of the local cancer clinical network. See Table 1. Physical signs, presented as photographs or descriptions, were available if the GP proposed relevant physical examination. The sixth scenario did not involve a cancer diagnosis. Actors were members of staff at a local research centre in Western Australia. They were instructed to present as patients consulting for ongoing care and to mention a new symptom during the consultation. See Table 2. A medical record was prepared for each patient and was available to the GP.

**Table 1:** Cancers

Diagnosis: Presenting as	
Session 1:	Session 2:
1. Lung cancer: haemoptysis	1. Colorectal cancer: Iron deficiency Anaemia
2. Non cancer patient: repeat prescription	2. Lung cancer: Smoker with stridor
3. Colorectal cancer: Diarrhoea and rectal bleeding	3. Breast cancer: Paget's disease presenting in the context of a history of atopic eczema
4. Colorectal cancer: Persistent diarrhoea	4. Lung cancer: Ex-smoker with cough and loss of appetite of 3 months duration
5. Breast cancer: Breast lump	5. Colorectal cancer: Diarrhoea and abdominal mass
6. Lung cancer: hoarseness, dyspnea, fatigue and unexplained weight loss	6. Non cancer patient: haemorrhoids

**Table 2:** Ticket of entry' to consult.

Session 1		Session 2	
**"Ongoing care" problem**	**Request or problem**	**"Ongoing care" problem**	**Request or problem**

Hypertension	Repeat prescription for antihypertension medication.	Angina	Uncontrolled angina presenting as requiring repeated use of nitrate spray
Hypertension	Repeat prescription for antihypertensive medication.	Injury to chest	Chest x-ray suggesting malignant pleural effusion.
Smoking	Advice to quit smoking.	Eczema	Rash
Diabetes	Routine referral to ophthalmologist	Sore throat	Antibiotics
Tennis elbow	Review of symptoms of tennis elbow.	Blood results	Results of full blood count suggesting iron deficiency anaemia.

### Innovation

**Figure 1:**
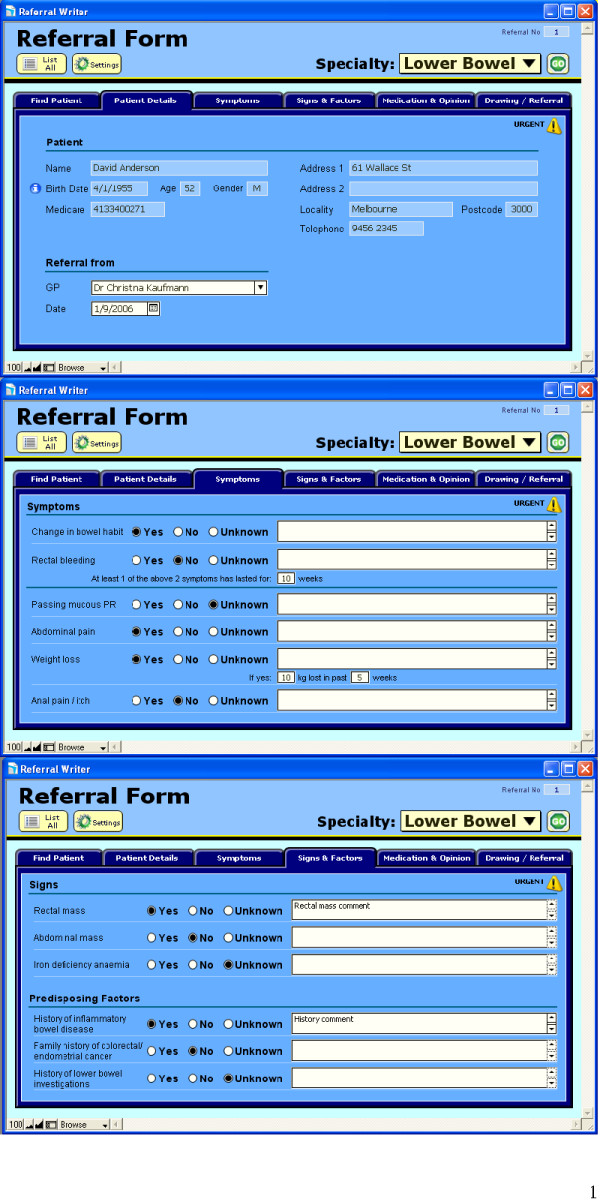
**Screen grabs for the 'referral writer' software**. Referrals to lower bowel specialist shown here.

**Figure 2:**
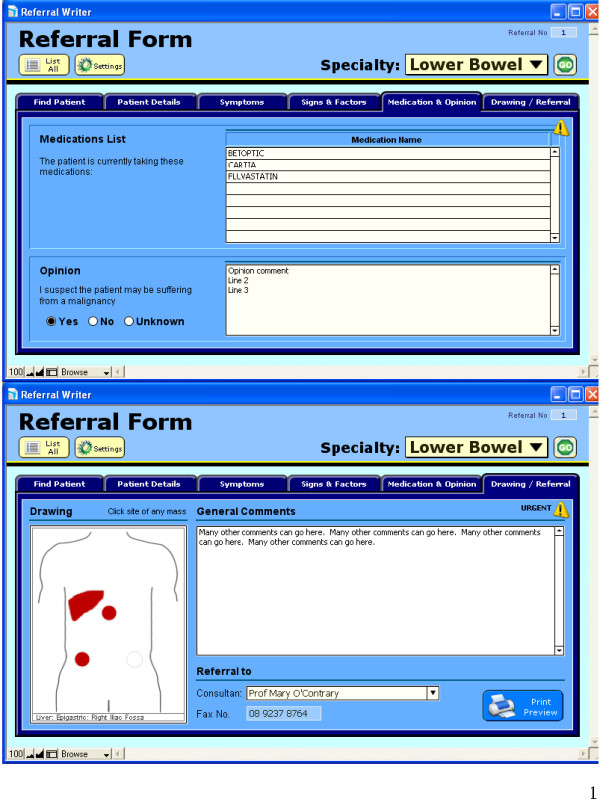
**Screen grabs for the 'referral writer' software**. Referrals to lower bowel specialist shown here.

An interactive referral pro forma was developed by a project team consisting of general practitioners and technical experts. The software was designed as an interactive referral letter which highlighted a 'red flag' presentation following algorithms based on recommended guidelines to one of three specialists: respiratory, colorectal and breast. A referral letter could be produced within two minutes and was intended to be used at the point where the practitioner had decided to make a referral. Screen grabs in relation to the colorectal referral are presented in Figure 1 and 2.

### Consultations

Six GP volunteers were asked to consult with the six actor-patients as though they had previously visited the practice for one or two ongoing medical problems (e.g. diabetes, hypertension etc). The practitioners were allocated 15 minutes per consultation. GPs were asked to make clinical notes and outline any management plan in as much detail as they would in their practice. GPs were informed that the study was about 'testing an innovation'. In the second set of consultations the practitioners were asked to deploy the software during the consultation if they decided to refer the patient. Video recordings were independently reviewed by two investigators (RMK and MJ).

### Outcome measures

Consultation competence: The LAP has been shown to facilitate reliable assessments of consultation performance and its face validity has been confirmed for general practice consultations. [7-9] Three of the six LAP categories of consultation competence (interviewing and history taking, problem solving and patient management) were assessed in this study. We double rated all available consultations and followed the methods described in the LAP and previous work on assessing recorded consultations [10-12] A difference of 5 or more in the LAP scores was considered 'clinically significant'. This was based on a standard deviation of about 10 for LAP assessments of 53 GPs and a before and after difference of 5 points (unpublished data from a series of studies on GPs' consultation skills). [13,14]

## Results

### Scoring by two assessors

Sixty of the seventy two consultations were recorded with sufficient sound and picture quality to allow analysis using the LAP. There were 60 consultations available for assessment, 36 before and 24 after the intervention. The distribution of consultations by intervention is shown in Table 3.

**Table 3:** Number of consultations available for assessment at each of the two sessions

GP id	Before intervention	After intervention	Total
1	6	0	6
2	6	6	12
3	6	6	12
4	6	6	12
5	6	3	9
6	6	3	9
**Total**	**36**	**24**	**60**

**Figure 3:**
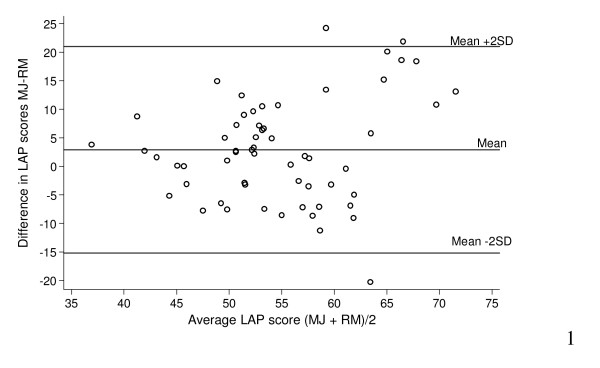
**Bland-Altman plot**.

Each video consultation was scored twice by two assessors leaving 120 LAP scores available for analysis. The mean difference in score between the two assessors was 2.9 (95% CI 0.6–5.3) with levels of agreement (+/- 2 SD) ranging from -15.2 to 21.0. This is illustrated in Figure 3. There was no evidence of a difference in the variance with increasing score (Pitmans test, P = 0.09). There was also no difference in the mean differences between assessor scores before and after the intervention (T-test, P = 0.49)

**Figure 4:**
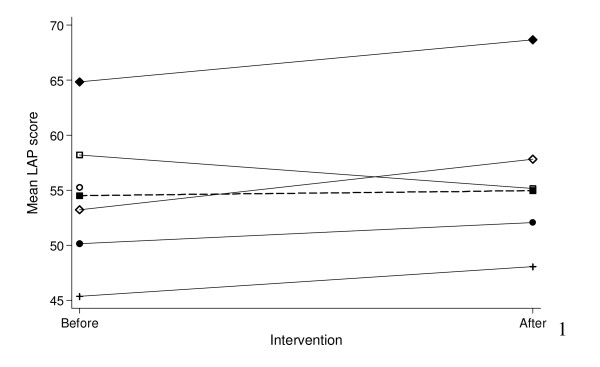
**Mean LAP score for each GP before and after the intervention**. The mean LAP score for the group before and after the intervention is shown by the dashed line. GP 1 had no after scores and is indicated by the open circle at before.

GEE modeling with main effects only indicated that there was no difference between mean LAP scores before and after the intervention and after taking clustering by GP and assessor into account (Mean difference 2.2, 95% CI: -0.9–5.4, p = 0.168). The type of scenario and use of the software was also not associated with a mean change in LAP score. However, an interaction between GP and intervention was observed in a subsequent GEE model which indicated that the effect of the intervention varied by GP (Table 4). This is also illustrated in Figure 4 where the mean LAP score increased for four GPs but decreased for one GP.

**Table 4:** Mean LAP scores estimated from GEE model that adjusted the standard errors for clustering by GP and Assessor and small sample bias. GP and intervention were entered into the model as an interaction term.

Variable	Mean difference in LAP score	95%CI	p-value
Assessor One	ref		
Assessor Two	2.3	-1.7–6.0	0.220
			
After vs. before intervention			
GP2	1.9	-7.1–10.9	0.679
GP 3	2.7	1.2–4.2	<0.001
GP 4	4.6	-3.6–12.8	0.272
GP 5	3.8	3.4–4.2	<0.001
GP 6	-3.1	-5.3–-0.8	0.008

Data from GP 1 was excluded from this intervention model.

## Discussion

The approach had several strengths; we were able to replicate conditions that may be difficult to control in clinical practice. The practitioners all consulted the same patients in the same practice on the same evening. In many ways the methodology involving consulting actor-patients mimics the formal assessment or examinations of candidates seeking membership to many professional colleges. This study however illustrates methodological and technical challenges of investigating the impact of innovations on consultations in this context.

### Medical practitioners and innovation

Participating GPs were volunteers and perhaps unrepresentative. That alone was not considered a major limitation in the design of this study which was intended to demonstrate the impact on the performance of volunteers in two stages. However we have no measures of how the volunteer practitioners perform in routine practice out with the study using the LAP or any other consultation competence measure. We are therefore unable to confirm how well their performance here reflects their competencies when consulting 'real' patients. We therefore recommend a preview of practitioner performance in routine practice or the development of a pool of practitioners available to test innovations in controlled conditions in order to provide a practitioner performance baseline. The data from this study also suggest that there was no significant harm in relation to the consultation competencies in the second phase of the study. In particular, we had hypothesized that this intervention could have disrupted the flow the consultations – the 'switch costs' of the intervention. [4,5] We had not direct measure of this although if this has been a problem we would have expected it to have been reflected in the LAP scores which were allocated. While this was encouraging and helpful data, we cannot exclude the possibility of a clinically important negative impact (a reduction of 5 scale points) of the intervention on the performance of three of the six GPs' consultations. Therefore we emphasize that this method is a prelude to, but not a substitute for, a formal randomised trial in clinical practice.

### The clinical challenge and the innovation

Many more people present to general practitioners with symptoms which could indicate cancer, than people who actually have cancer: symptoms have a relatively low sensitivity and specificity. Conversely, forty percent of cancer patients reported significant problems communicating their concerns in the pre-diagnostic period and needed recurrent GP consultations before being 'taken seriously'.[15] We hypothesized that the administrative tool may have heightened the practitioners inattentiveness to other important clinical issues and reduced the LAP scores. We therefore introduced the innovation to consultations, where cancer featured prominently on the list of differential diagnosis. In practice however the clinical encounters adopted for this study were fairly self evident. Indeed the GPs commented afterwards that the majority of the consultations involved cancer symptoms, which was 'unusual' in reality and may have affected their performance. Neither practitioner examination skills nor the impact of that examination could be assessed in this experiment. However it may be impractical and possibly unethical to subject actors to intimate physical examination. In this study it is also possible that there were subtle differences in the style of presentation which may have had an impact on practitioner performance. The employment of professional actors may have been an advantage. It may also be important that the study is conducted in a setting that more closely resemble the practitioner's own rooms. It may be necessary to furnish the study 'clinic room' and arrange the furniture by seeking the participating practitioners' preferences.

### The scoring of consultation competencies

Recording of consultations needs to be facilitated by technicians guaranteeing high quality footage and with the least disruption or inconvenience to the participants. Unfortunately a proportion of consultation was not captured on tape due to technical failures and so could not be analysed. It may be helpful in a repeat of this study to employ a professional media team to facilitate the recordings. We were unable to assess the impact of observation on the GPs' performance although the literature on video recording for the purposes of assessment suggests that it has no significant adverse effect. [16] Agreement by assessors on GP LAP scores was generally good. The assessors were from similar practice backgrounds (UK) and seniority as GPs (20 vs. 15 years), but differed in experience of assessment (15 vs. 3 years) and familiarity with the LAP (RMK involved in the design, development and evaluation of the LAP, MJ new to it) More resources need to be devoted to cross-training and calibration of the assessors. Nevertheless, we did not record any significant difference in the assessment of cases with reference to impact of the innovation. However, this study was designed to investigate the practicalities of establishing the methodology rather than obtain conclusive results in relation to a hypothesis. Finally as investigators in the study the assessors could not be blinded to aims of the study. We do not believe this had an impact on the scores however it would be prudent to deploy assessors who could be successfully blinded at the time of reviewing the consultations.

## Conclusion

Several important lessons were learned in relation to testing innovations in 'controlled conditions'. The design of a 'clinical laboratory' in general practice, focusing on the consultation, requires painstaking attention to details such as the performance of the technical equipment, the actors, the practitioners and the assessors. There is a risk of significant failures in all aspects, each having a bearing on the validity of the results. In this study we offer some preliminary ideas on the design of such a facility with respect to testing innovations as prelude to a formal clinical trial in general practice.

## Competing interests

The authors declare that they have no competing interests.

## Authors' contributions

MJ, RMcK, designed the study and co-authored the paper, KS analysed the data and co-authored the paper, HA and MS organized the simulated consultations, collected the data and co-authored the paper. All authors read and approved the manuscript.

## Pre-publication history

The pre-publication history for this paper can be accessed here:

http://www.biomedcentral.com/1471-2288/9/54/prepub
